# Luteolin inhibits triple-negative breast cancer by inducing apoptosis and autophagy through SGK1-FOXO3a-BNIP3 signaling

**DOI:** 10.3389/fphar.2023.1200843

**Published:** 2023-06-06

**Authors:** Ling Wu, Yingda Lin, Songyu Gao, Yongfang Wang, Huiji Pan, Zhaozhi Wang, Marina Pozzolini, Fengling Yang, Haiyan Zhang, Yi Yang, Liang Xiao, Yuan Xu

**Affiliations:** ^1^ Medical College of Yangzhou University, Yangzhou, China; ^2^ Department of Pharmacy, The Affiliated Hospital of Yangzhou University, Yangzhou University, Yangzhou, China; ^3^ Dongzhimen Hospital, Beijing University of Chinese Medicine, Beijing, China; ^4^ Faculty of Naval Medicine, Second Military Medical University (Naval Medical University), Shanghai, China; ^5^ Department of Occupational and Environmental Health, Xiangya School of Public Health, Central South University, Changsha, China; ^6^ School of Pharmacy, Xinjiang Medical University, Urumqi, China; ^7^ Department of Earth, Environment and Life Sciences (DISTAV), University of Genova, Genova, Italy; ^8^ College of Traditional Chinese Medicine, Jilin Agricultural University, Changchun, China; ^9^ Department of Healthcare, Changhai Hospital, Second Military Medical University (Naval Medical University), Shanghai, China; ^10^ Department of Cardiology, The Affiliated Hospital of Yangzhou University, Yangzhou University, Yangzhou, China

**Keywords:** triple-negative breast cancer, luteolin, SGK1, BNIP3, apoptosis, autophagy

## Abstract

**Background:** Triple-negative breast cancer (TNBC) is one of the most prominent neoplasm disorders and lacks efficacious treatments yet. Luteolin (3′,4′,5,7-tetrahydroxyflavone), a natural flavonoid commonly presented in plants, has been reported to delay the progression of TNBC. However, the precise mechanism is still elusive. We aimed to elucidate the inhibition and molecular regulation mechanism of luteolin on TNBC.

**Methods:** The effects of luteolin on the biological functions of TNBC cells were first evaluated using the corresponding assays for cell counting kit-8 assay, flow cytometry, wound-healing assay, and transwell migration assay, respectively. The mechanism of luteolin on TNBC cells was then analyzed by RNA sequencing and verified by RT-qPCR, Western blot, transmission electron microscopy, etc. Finally, *in vivo* mouse tumor models were constructed to further confirm the effects of luteolin on TNBC*.*

**Results:** Luteolin dramatically suppressed cell proliferation, invasion, and migration while favoring cell apoptosis in a dose- and time-dependent manner. In TNBC cells treated with luteolin, SGK1 and AKT3 were significantly downregulated while their downstream gene BNIP3 was upregulated. According to the results of 3D modeling, the direct binding of luteolin to SGK1 was superior to that of AKT3. The inhibition of SGK1 promoted FOXO3a translocation into the nucleus and led to the transcription of BNIP3 both *in vitro* and *in vivo*, eventually facilitating the interaction between BNIP3 and apoptosis and autophagy protein. Furthermore, the upregulation of SGK1, induced by luteolin, attenuated the apoptosis and autophagy of the TNBC.

**Conclusion:** Luteolin inhibits TNBC by inducing apoptosis and autophagy through SGK1-FOXO3a-BNIP3 signaling.

## Graphical Abstract

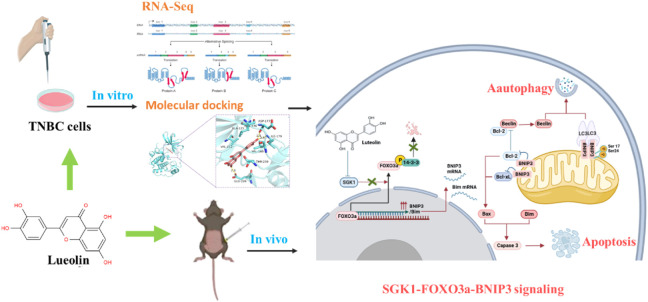

## Introduction

As a special type of breast cancer, which has had the highest incidence and mortality rates since 2020 (Sung et al., 2021), triple-negative breast cancer (TNBC) is named for the three hormone receptors it lacks-estrogen receptor-negative (ER^−^), progesterone receptor-negative (PR^−^), and human epidermal growth factor receptor-2 negative (HER2^−^) (Font-Clos et al., 2022), which restricts its interventions and therapeutic strategies. Currently, the standard strategy for treating TNBC is chemotherapy with paclitaxel/anthracycline-containing agents (Lee et al., 2020). Nevertheless, severe toxic effects, lack of distinct molecular targets, and drug resistance drastically lead this treatment to low overall survival probability and poor prognosis of TNBC patients (Vasan et al., 2019). Therefore, the excavation of innovative and efficacious alternative medications with few adverse reactions and freshly precise biomarkers for TNBC is urgently required.

Natural products and their derivatives have already been an abundant source to develop novel small-molecule compounds in cancer therapy (Li and Weng, 2017), among which herbs have been used to extract small-molecule compounds for cancer therapy. One of the most researched phytochemicals, flavone luteolin (3’,4’,5,7-tetrahydroxyflavone), exists in a wide and diverse range of vegetables, medicinal herbs, flowers, and spices (Yashin et al., 2017; Gendrisch et al., 2021; Caporali et al., 2022), has been extensively used to fight against breast cancer (Ahmed et al., 2019; Fasoulakis et al., 2021), colorectal cancer (Ambasta et al., 2018), lung cancer (Pan et al., 2022), and prostate cancer models (Ye et al., 2021) over the past several decades, by disarraying the cell cycle progression, suppressing proliferation, migration, invasion, and stimulating apoptosis of cancer cells. Han-Tsang Wu and others reported that luteolin controlled the expression of MMP9 to prevent the growth and metastasis of androgen receptor-positive TNBC (Wu et al., 2021). Lin et al. (2017) also indicated that luteolin successfully prevented TNBC metastases by reversing EMT, which may be caused by β-catenin downregulation. However, few studies are focusing on the molecular target and sequence signaling cascade activity in the regulation of luteolin on TNBC progression.

Apoptosis is a highly organized and orchestrated programmed cell death process that occurs in physiological and pathological circumstances, associated with the eradication of potentially malignant cells, hyperplasia, and tumor progression (Kerr et al., 1972). In addition, autophagy, as a strictly controlled catabolic progress, plays a dual function in the emergence and development of cancer (Kimmelman and White, 2017). Autophagy and apoptosis have a close association and they could be activated by common upstream signals during the process of cancer cell death (Su et al., 2015; Chen et al., 2018). Even though it has been concluded in various studies that luteolin exerts anticancer potential via eliciting both apoptosis and autophagy (Ashrafizadeh et al., 2020; Chen et al., 2023; Kulaphisit et al., 2023; Li et al., 2023), the precise mechanisms have not been identified, particularly in TNBC.

In this study, we examined the effects of luteolin on proliferation, apoptosis, migration, invasion, and gene expressions on TNBC cells *in vitro and* its anti-TNBC effects *in vivo*, to assess its therapeutic potential on TNBC and investigate its putative molecular mechanisms.

## Material and methods

### Cell culture and reagents

The TNBC cell lines MDA-MB-231 and 4T1 cells were commercially obtained from Procell Co., Ltd. (Wuhan, China). Cells were maintained in DMEM medium supplemented with 10% FBS containing penicillin and streptomycin in the incubator of 5% CO^2^ at 37°C. Luteolin (purity, ≥98%; molecular weight, 286.24; formula, C_15_H_10_O_6_) was purchased from Baoji Herbest Biotechnology Corporation (Shanxi, China). Luteolin was prepared at 50 mM by dissolving in dimethyl sulfoxide (DMSO) (Solarbio, Beijing, China) and stored at −20°C.

### Cell proliferation and apoptosis assay

TNBC cells (1 × 10^4^ cells/well) were plated in 96-well plates and given luteolin treatment with different concentrations (0, 12.5, 25, 50, 100, 200 μM) to track cell proliferation using CCK-8 assay at 12, 24, 48, and 72 h. CCK-8 reagent (TopScience, Shanghai, China) was added and the absorbance was recorded at 450 nm. To quantify apoptosis, TNBC cells were exposed to luteolin for 24 and 48 h in 6-well plates and collected to examine with Annexin V-FITC/PI kit (BD Pharmingen, CA, United States). 100 μL of the cells solution (1 × 10^5^ cells) was mixed with 5 μL Annexin V-FITC and 5 μL propidium iodide being incubated for 15 min at room temperature (25°C) in the dark, then the labeled TNBC cells were monitored using flow cytometry.

### Cell migration and invasion assays

The motility status of TNBC cells was assessed by Wound-healing assay and Transwell assays.

In the cell monolayer, a wound was made with a sterile 200 L plastic pipette tip before fresh media with different luteolin concentrations was added. A total of 1 × 10^5^ TNBC cells treated with luteolin were cultivated in upper chambers (Corning Costar, NY, United States) with serum-free medium covered with or without Matrigel and treated with luteolin (BD Bioscience, CA, United States), while media supplemented with 10% FBS was added to the lower chambers. Next, fixed TNBC cells with 4% paraformaldehyde were dyed with 0.5% crystal violet after incubation for 48 h. Photomicrographs of wound closure and the number of TNBC cells that passed through the chambers were taken by a microscope.

### Bioinformatics analysis

We compared the gene expression levels of MDA-MB-231 Cells with and without luteolin by performing RNA sequencing (RNA-seq). MDA-MB-231 cells were harvested after treatment with 50 μM luteolin or control (DMSO) for 48 h in triplicate, and stored at −80°C for RNA-seq analysis (LC-Bio Technology CO., Ltd., Hangzhou, China). NanoDrop ND-1000 (NanoDrop, Wilmington, DE, United States) was used to measure the quantity and purity of RNA in each sample. Then, using the Magnesium RNA Fragmentation Module (NEB, cat.e6150, United States), the poly(A) RNA was fragmented into small pieces. Reverse transcription was carried out on the cleaved RNA fragments by SuperScriptTM II Reverse Transcriptase (Invitrogen, cat.1896649, United States) to create the cDNA, which was then utilized to create second-stranded DNAs that were U-labeled. The ligated products are amplified by PCR following treatment with the heat-labile UDG enzyme (NEB, cat.m0280, United States). The resultant cDNA collection had an average insert size of 300 ± 50 bp. At last, the Illumina NovaseqTM 6000 (LC-Bio Technology CO., Ltd., Hangzhou, China) was used to carry out the 2150 bp paired-end sequencing (PE150) following the vendor’s recommended protocol. The differentially expressed gene (DEGs) were selected and the R package subsequently performed GO enrichment and KEGG enrichment analysis. Venn diagrams and a cluster heat map of DEGs were generated in the web platform produced by LC-Bio Technology CO., Ltd. The interaction among the proteins of DEGs was determined by using the STRING database (https://string-db.org/).

### Molecular docking

According to the CAS number, the 3D structure of luteolin was downloaded in SDF format from PubChem data and imported into ChemBio3D Ultra 14.0 for energy minimization. Small molecules were saved in mol2 format with a minimum root-mean-square gradient of 0.001. AutodockTools-1.5.6 was used to optimize small molecules for hydrogenation, calculating the charge, assigning the charge, setting the rotatable bond, and saving it as “pdbqt” format. AKT3 (PDB ID:2X18) and SGK1 (PDB ID:2R5T) protein structures were downloaded from the PDB database. Crystal water and the original ligands were eliminated using Pymol 2.3.0 software. Potential ligand-binding sites were predicted using POCASA 1.1 and the molecular docking was executed by AutoDock Vina 1.1.2. Maestro 11.8 and PyMOL2.3.0 were used to visually examine the ideal docking stance.

### RT-qPCR

RT-qPCR was used to verify whether the gene expression changes of MDA-MB-231 cells treated with luteolin are the same as the results of RNA sequencing to estimate the veracity of bioinformatic analysis. The TRizol reagent (Invitrogen) was used to separate the total RNA from MDA-MB-231 cells. Then the total RNA was reversed to cDNA according to the manufacturer’s instructions with TaqMan microRNA reverse transcription kit (Toyobo, Osaka, Japan). The cDNA was used for RT-qPCR with specific gene primers (Table 1) using SYBR-green (TopScience, Shanghai, China). The relative expression of genes was calculated using the 2^−ΔΔCT^ method with GAPDH serving as an internal reference and normalization.

**TABLE 1:** The primer sequences amplified used for RT-PCR.

Gene	Sequences
SGK1-forward	5′ -CAT​ATT​ATG​TCG​GAG​CGG​AAT​GT-3′
SGK1-reverse	5′ -TGT​CAG​CAG​TCT​GGA​AAG​AGA -3′
IL6-forward	5′ -ACT​CAC​CTC​TTC​AGA​ACG​AAT​TG-3′
IL6-reverse	5′ -CCA​TCT​TTG​GAA​GGT​TCA​GGT​TG-3′
CDKN1A-forward	5′ -TGT​CCG​TCA​GAA​CCC​ATG​C-3′
CDKN1A-reverse	5′ -AAA​GTC​GAA​GTT​CCA​TCG​CTC-3′
EGF-forward	5′ -TGT​CCA​CGC​AAT​GTG​TCT​GAA-3′
EGF-reverse	5′ -CAT​TAT​CGG​GTG​AGG​AAC​AAC​C-3′
AKT3-forward	5′ -TGT​GGA​TTT​ACC​TTA​TCC​CCT​CA-3′
AKT3-reverse	5′ -GTT​TGG​CTT​TGG​TCG​TTC​TGT-3′
IL7R-forward	5′ -TGT​CGT​CTA​TCG​GGA​AGG​AG-3′
IL7R-reverse	5′ -CGG​TAA​GCT​ACA​TCG​TGC​ATT​A-3′
GAPDH-forward	5′ -GGA​GCG​AGA​TCC​CTC​CAA​AAT-3′
GAPDH-reverse	5′ -GGC​TGT​TTC​ACT​TCT​CTC​ATG​G-3′

### Western blot

Proteins were extracted from cells and tissues by protein extraction reagent SDS Lysis Buffer (Beyotime, Shanghai, China) and the samples were centrifuged at 10,000 *g* for 15 min at 4°C to collect the supernatant. With the use of the BCA protein assay kit (Beyotime, Shanghai, China), the protein concentration was determined and the protein was separated by 12.5% SDS-PAGE to transfer into PVDF membranes. The membranes were blocked for 2 h at room temperature (25°C) and incubated with different SGK1 (Abcam, Ab32374), FOXO3a (ProteinTech, 10849-1-AP), p-FOXO3a (Affinity, AF3020), BNIP3 (ProteinTech, 68091-1-Ig), Bcl-2 (ProteinTech, 26593-1-AP), Bax (ProteinTech, 50599-2-Ig), Bim (ProteinTech, 22037-1-AP), LC3 (Abcam, ab192890), Beclin-1 (Cell Signaling Technology, 3738S), p62 (ProteinTech, 18420-1-AP), and GAPDH (Santa Cruz, sc-47724) antibody overnight at 4°C. After incubation with the primary antibody, the secondary antibody was followed for 2 h at room temperature (25°C) and washed with TBST. Finally, the results were visualized by using film exposure with an ECL substrate. Relative expression was quantified with ImageJ and GAPDH was used as an internal control.

### Transmission electron microscopy

MDA-MB-231 cells were collected and fixed with 2.5% glutaraldehyde overnight before being wrapped in 1% agarose following a 48-h treatment with 50 M luteolin. Then it was processed in the following steps: wash the cell centrifugation with 0.1 M phosphate buffer PB (PH7.4) 3 times and wrap it with 1% agarose solution. The sample was then fixed with a 1% osmium acid solution for 2 h followed by room temperature dehydration. Following this, the cells underwent treatments with 1:1 acetone:812 embedding medium for 2–4 h, 1:2 acetone:812 embedding medium infiltration overnight, and pure 812 embedding medium for 5–8 h. Following the completion of the polymerization, the cells were dissolved in a 40% hydrofluoric acid solution for 15 min before being polymerized once more. The resin blocks were taken out of the embedding plate and cut to 60–80 nm thin on the ultramicrotome after 48 h of polymerization in a 60°C oven. Then the samples were then spread out onto the 150 meshes cup rum grids with formvar film, stained for 8 min with a 2% uranium acetate saturated alcohol solution, and rinsed three times in 70% ethanol. Images of stained sections were observed using TEM by Servicebio (Wuhan, China).

### Vector construction and transfection

To validate the SGK1 activity in the action of luteolin, full-length cDNA of the human SGK1 gene was entirely synthesized and placed into Vector plasmid pCDNA3.1 (+) using the In-Fusion cloning system according to the manufacturer’s protocols (GENEWIZ, Suzhou, China) to construct pCDNA-SGK1. MDA-MB-231 cells were seeded into a 6-well cell culture plate, and the transfection was performed at 70% cell density. The vector pCDNA3.1 (+) which was used as controls and pCDNA-SGK1 were transfected respectively into MDA-MB-231 cells with Lip8000 (Beyotime, Shanghai, China). For transfection, 4 μL of Lipo8000™ reagent was suspended in 125 μL of serum-free medium and 2.5 μg of each pCDNA3.1 (+) and pCDNA-SGK1. Overexpression of the SGK1 was verified by qRT-PCR and Western blot analysis to carry out the follow-up experiment.

### Mice tumor models

Animal experiments for care and use were strictly conducted under institutional ethical guidelines and the Animal Care and Use Committee of Naval Medical University (Shanghai, China). Five-week-old female BALB/c mice (average body weight 17–20 g) were housed under specific pathogen-free conditions and fed with sterilized food and water. On day 0, each mouse was implanted subcutaneously with viable 5 × 10^5^ 4T1 cells in the right mammary fat pad to establish primary model tumors. Eighteen mice were haphazardly selected and addressed separately into three groups (six mice in each group) to receive treatment on day 7. Mice of each group were injected with 10% DMSO, 20 mg/kg, and 40 mg/kg luteolin every day respectively. The weight and size of the tumor were measured once in 2 days and calculated by a standard formula: volume = length × (width)^2^/2. At the end of both experimental periods (21 days after treatments), mice were sacrificed and tumors were dissected and weighed. The tumor tissue is either stored at −80°C or preserved in formalin for further analysis.

### Hematoxylin-eosin staining and immunocytochemistry

The animal tumor tissue was removed and fixed in 10% formaldehyde for at least 24 h. Then the tissues were fixed in paraffin solution and slices of tumor tissue measuring 4 μm thick were cut and mounted on a glass slide. Hematoxylin and eosin (H&E) solutions were employed to stain the slices and the H&E-stained images were evaluated to search for tumor structural alterations in response to luteolin under a microscope. To determine the changes in apoptosis and autophagy-related gene expression, immunocytochemistry was performed as reported. The 3% hydrogen peroxide was used to quench the endogenous peroxidase activity of paraffin slices and 3% BSA was used to block tissue. Then the section was incubated with primary antibodies against SGK1, FOxO3a, BNIP3, and LC3 at 4°C overnight and followed incubated with a secondary antibody for 2 h. Finally, the slices were thoroughly washed in PBS, and diaminobenzidine was added to visualize the slices. The same procedures were used for the control sections, but no primary antibodies were used. The images were captured by microscope.

### Statistics

All the presented data and outcomes were verified by at least three separate experiments. The experimental data were expressed as means ± SEM and analyzed using GraphPad Prism 8.0. Statistical differences between the two groups were evaluated with a *t*-test, while A one-way analysis of variance (ANOVA) was used to assess statistical differences between numerous groups, while a *t*-test was used to assess statistical differences between two groups. *p* < 0.05 was considered statistically significant.

## Results

### Luteolin represses the viability and promotes the apoptosis of TNBC cells

**FIGURE 1:**
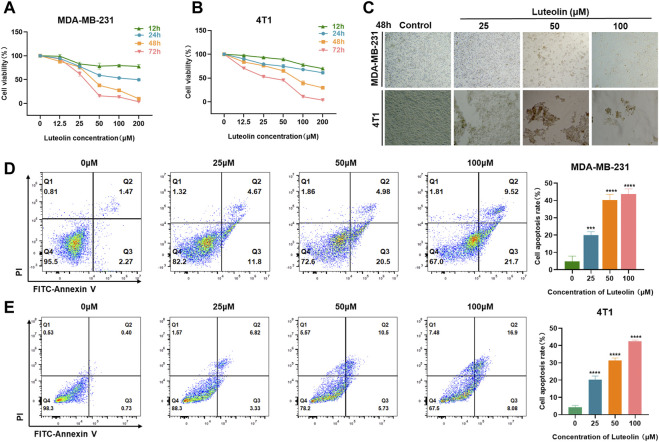
Luteolin inhibited triple-negative breast cancer cell viability and promoted apoptosis. **(A,B)** Luteolin significantly inhibited the cell viability of MDA-MB-231 cells **(A)** and 4T1 cells **(B)** in a time and dose-dependent manner. **(C)** The morphological features of MDA-MB-231 and 4T1 cells treated with luteolin. **(D,E)** Apoptosis activity of MDA-MB-231 cells **(D)** and 4T1 cells **(E)** treated with luteolin for 48 h. All results are represented as the mean ± SEM for at least three independent experiments. *p < 0.05, **p < 0.01, ***p < 0.001, ****p < 0.0001.

To verify the effects of luteolin on the viability and apoptosis of TNBC cells, CCK-8 assays were performed in different doses and intervention duration. The results showed that luteolin in the concentration of 25 μM had a modest influence while 50 μM luteolin had significant inhibitory activity at 48 and 72 h, indicating a cell proliferation inhibiting effect in a dose and time-depending manner (Figures 1A, B). The half-maximal inhibitory concentration values of luteolin treated for 48 h were 39.31 μM for MDA-MB-231 cells and 63.06 μM for 4T1 cells (Supplementary Figures S1A, B). Furthermore, luteolin induced typical apoptosis morphological changes, like shrinkage, irregular shape, reduced density, and detachment in TNBC cells (Figure 1C). In addition, the strict cell joints were severely destroyed as the concentration of luteolin increased and the lapse of time (Supplementary Figures S1C, D). An Annexin V-FITC/PI experiment additionally validated that luteolin did noticeably promote apoptosis in MDA-MB-231 cells and 4T1 cells (Figures 1D, E). The total percentage including early and late apoptotic cells raised 12.73% in MDA-MB-231 cells and 9.02% in 4T1 cells with the treatment of 25 μM luteolin. And the ratio increased in a dosed manner, and the apoptosis rate increased by 27.48% in MDA-MB-231 cells and 23.85% in 4T1 cells when the drug concentration was 100 μM. Besides it, the apoptosis ratio at 48 h was significantly higher than that at 24 h (Supplementary Figures S1E, F). These findings suggest that luteolin could effectively repress the viability and promote the apoptosis of TNBC cells.

### Luteolin inhibits the invasion and migration of TNBC cells

**FIGURE 2:**
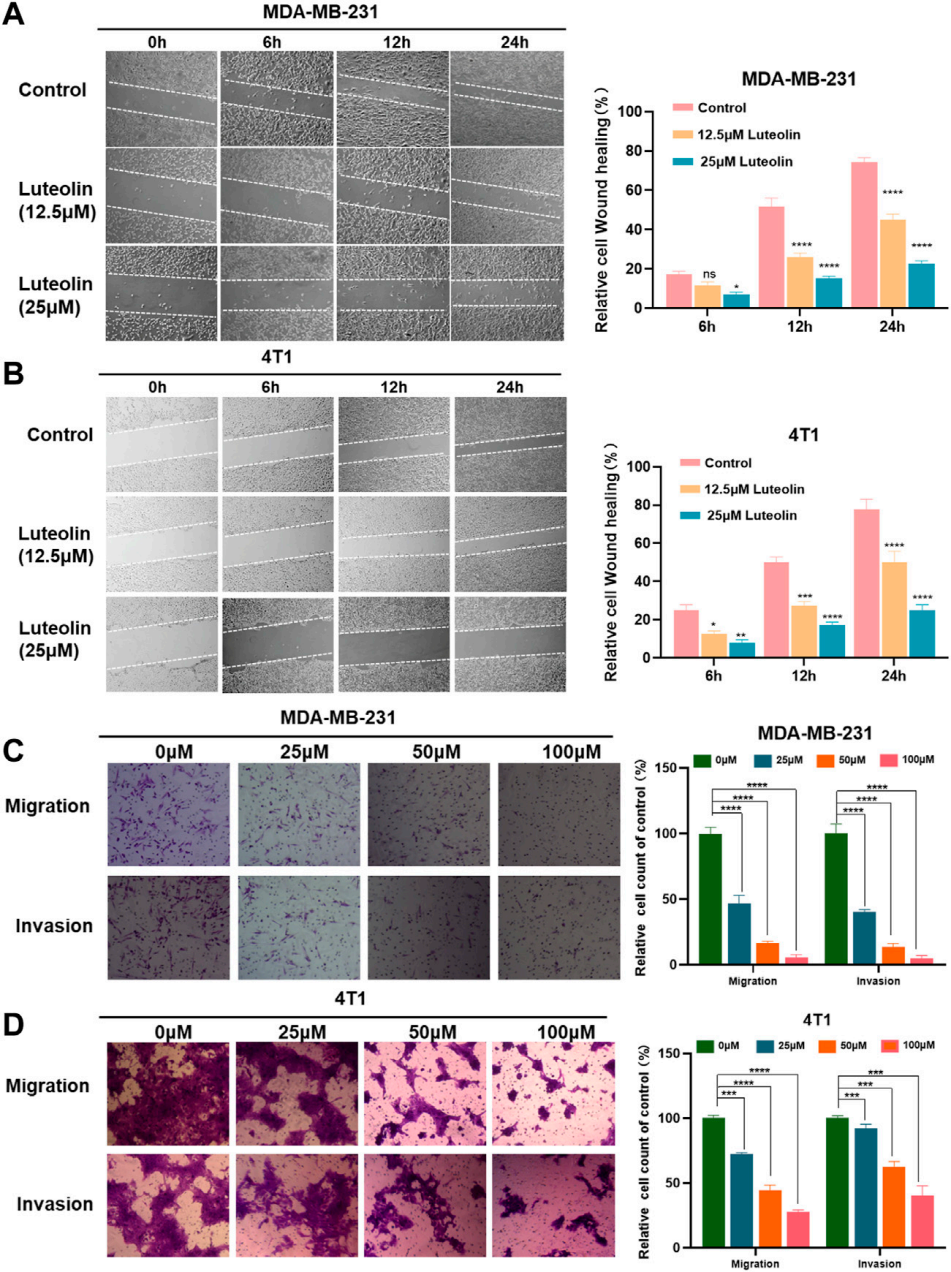
Luteolin suppressed the migration and invasion of triple-negative breast cancer cells. **(A,B)** Analysis of the migration activity of MDA-MB-231 cells **(A)** and 4T1 cells **(B)** treated with luteolin by Wound -healing assay. **(C,D)** Analysis of migration and invasion activity of luteolin-treated MDA-MB-231 cells **(C)** and 4T1 cells **(D)** by Transwell assay. All results are represented as the mean ± SEM for at least three independent experiments. *p < 0.05, **p < 0.01, ***p < 0.001, ****p < 0.0001.

Utilizing this wound-healing experiment, we demonstrated that both 4T1 and MDA-MB-231 treated with luteolin had a lower wound closure ratio in a dose-dependent manner (Figures 2A, B). To eliminate the interference of cytotoxic effects of luteolin on the cell lines, we chose low concentrations of luteolin for scratch experiments and carry out the experiments within 24 h. The results showed that the cells in the control group showed very fast healing. On the contrary, the addition of luteolin significantly decreased cell migration activity, and the scratch remained open after 24 h. The inhibition of migration by luteolin is in a time-dependent and dose-dependent way.

Similarly, the results of the transwell assay showed that luteolin decreased the migration and invasion abilities of TNBC cells. As shown in Figures 2C, D, 50 μM luteolin markedly reduced the migrated and invaded TNBC cells and the cells almost lost the ability to pass through the membrane of the chamber after being treated with 100 μM luteolin. This evidence collectively indicated that luteolin possesses anti-migration or invasion capabilities toward TNBC cells.

### RNA sequencing analysis identified downstream targets of luteolin

**FIGURE 3:**
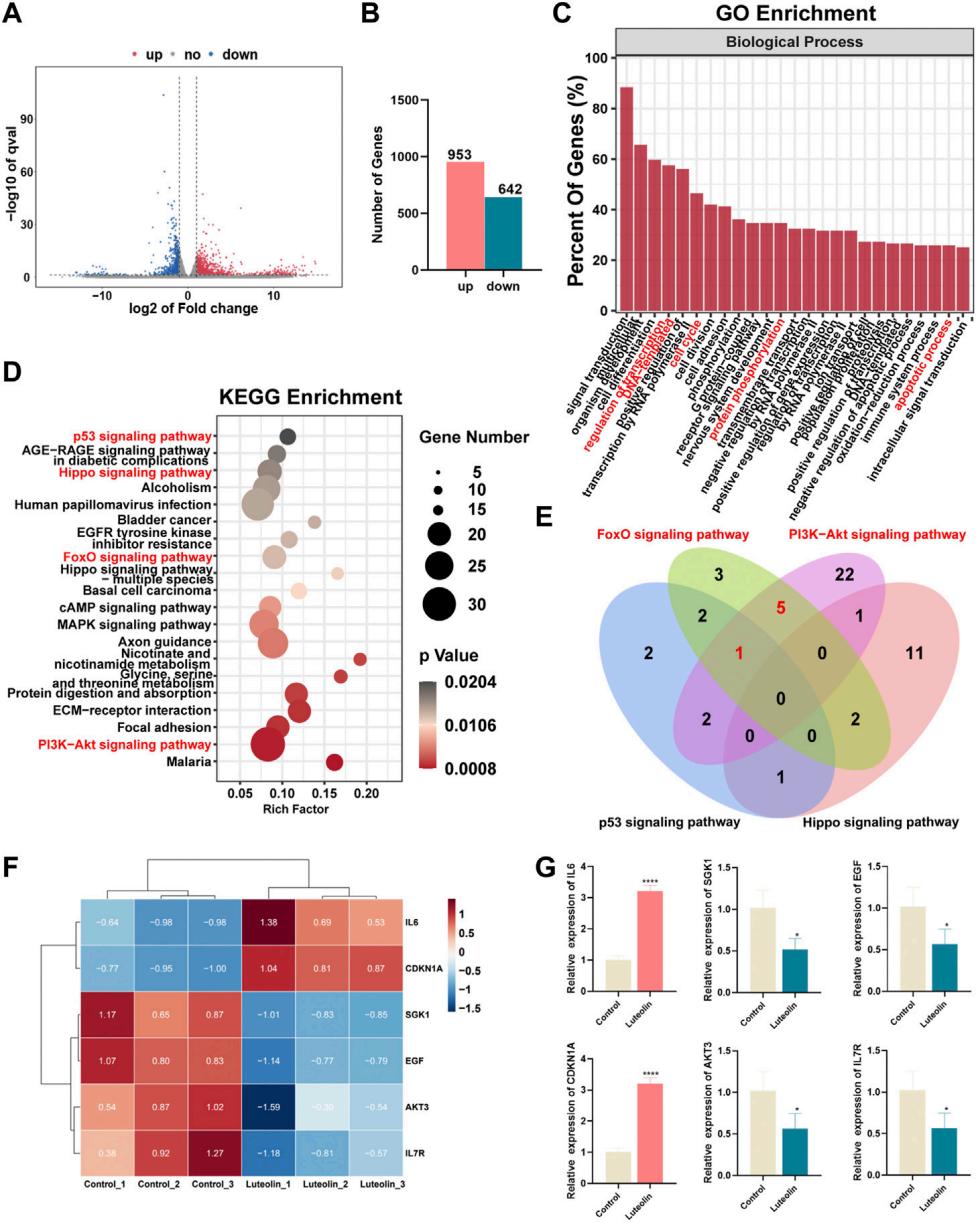
The effect of luteolin on the triple-negative breast cancer cell is associated with PI3K-AkT and FoXO signaling pathways. **(A)** Volcano plot displaying significantly differentially expressed genes (DEGs). Red plots represent the upregulated genes and blue plots represent the downregulated genes. **(B)** The number of DEGs. **(C)** GO analysis of DEGs. **(D)** KEGG analysis of DEGs. **(E)** The crosstalk of DEGs among p53, Hippo, PI3K-AkT, and FoXO signaling pathways. **(F)** Heatmap of PI3K-AkTand FoXO-related DEGs. **(F)** The mRNA expression of crosstalk DEGs among PI3K-AkT and FoXO signaling pathway. All results are represented as the mean ± SEM for at least three independent experiments. **(G)** The mRNA expression of crosstalk DEGs among PI3K-AkT and FoXO signaling pathway. *p < 0.05, **p < 0.01, ***p < 0.001, ****p < 0.0001.

RNA sequencing analysis was performed to seek out altered gene expression in MDA-MB-231 cells and gain insight into the molecular mechanism (Supplementary Table S1). Based on the threshold of |log2FC|≥1 & *q* < 0.05, A significant amount of differentially expressed genes between the two groups were discovered (Figure 3A), consisting of 953 upregulated and 642 downregulated genes (Figure 3B). According to gene ontology (GO) analysis, luteolin dramatically affected the DEGs in terms of regulation of transcription, cell cycle, protein phosphorylation, and apoptotic process in biological processes (Figure 3C). Furthermore, the Kyoto Encyclopedia of Genes and Genomes (KEGG) analysis demonstrated that DEGs significantly enriched in the signal pathways associated with cell cycle and apoptosis progress, namely, the p53 signaling pathway, Hippo signaling pathway, FOXO signaling pathway, and PI3K-Akt signaling pathway (Figure 3D). Noteworthy, six genes with significant expression level changes were repeated in the FOXO signaling pathway and PI3K-Akt signaling pathway, emphasizing considerable crosstalk between these two pathways (Figure 3E). Among them, IL6 and CDKN1A were upregulated, while SGK1, EGF, AKT3, and IL7R were downregulated (Figure 3F). And the mRNA expression level in these six genes was in agreement with RNA sequencing results (Figure 3G).

### The predicted binding mode of luteolin and SGK1/AKT3

**FIGURE 4:**
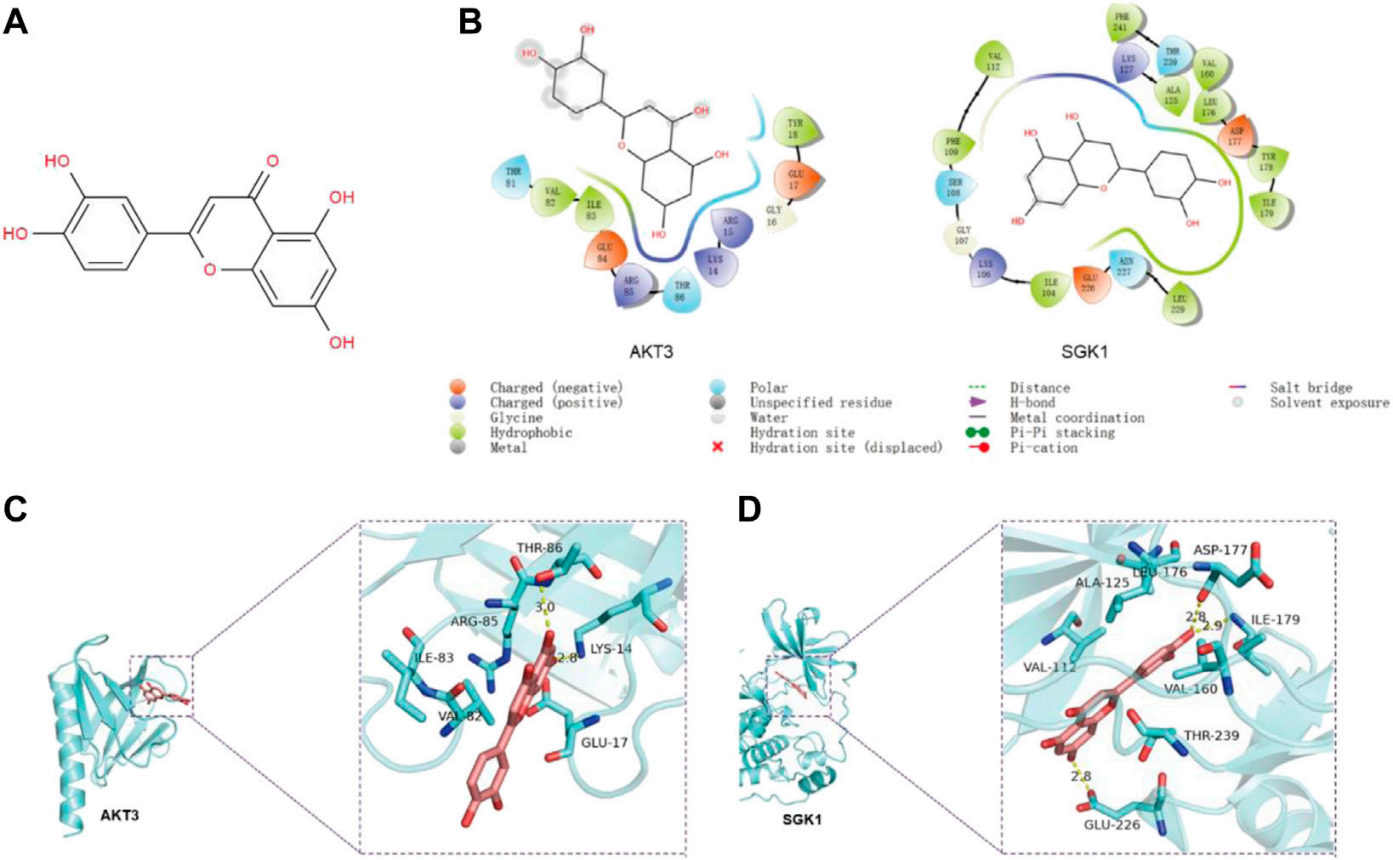
The predicted binding mode of luteolin and SGK1/AKT3. **(A)** Molecular structure of luteolin. **(B)** The binding modes between luteolin and AKT3/SGK1 generated by Maestro. **(C–D)** The 3D structure models of the luteolin–SGK1/AKT3 complex.

The results of the program AutoDock Vina1.1.2 showed that the complex of luteolin (Figure 4A) and SGK1 was more stable (with a binding energy of −9.1 kcal/mol) than AKT3 (with a binding energy of −7.3 kcal/mol). The analysis of binding modes by Maestro indicates that the complexes were stabilized by polar interaction and hydrogen bond interactions. Luteolin has polar interactions with SER108, ASN227 and THR239 and hydrophobic interactions with VAL-112, PHE109, ILE104, LEU229, ILE-179, TYR178, LEU176, VAL160, ALA125, and PHE241 of SGK1 (Figure 4B). As for AKT3, Luteolin mainly has polar interaction with THR81 and THR86 and hydrophobic interaction with VAL-82, ILE-83, and TYR1 (Figure 4B). The three-dimensional structure models generated by PyMol showed that the main forms of interactions between luteolin and AKT3/SGK1 were hydrogen bonds and hydrophobic forces. Luteolin forms a hydrogen bond with THR-86 and LYS-14 of AKT3, with bond lengths of 3.0Å and 2.8Å, respectively, and has a hydrophobic effect with GLU-17, ARG-85 ILE-83, and VAL-8 (Figure 4C). Luteolin interacts with SGK1 mainly through hydrogen bonds with GLU-226, ILE-179, and ASP-177, woth bond longths of 2.8Å, 2.9Å, and 2.8Å, respectively, and has hydrophobic interactions with THR-239, VAL-160, LEU1-76, ALA-125, and VAL-111 (Figure 4D). These results suggested that luteolin has a certain interaction with AKT3 and SGK1 of PI3K-AKT and FOXO pathway, and better with SGK1. Thus, we selected SGK1 as a candidate target for further investigation.

### Luteolin promotes apoptosis and autophagy in TNBC cells via the SGK1-FOXO3a-BNIP3 signaling pathway

**FIGURE 5:**
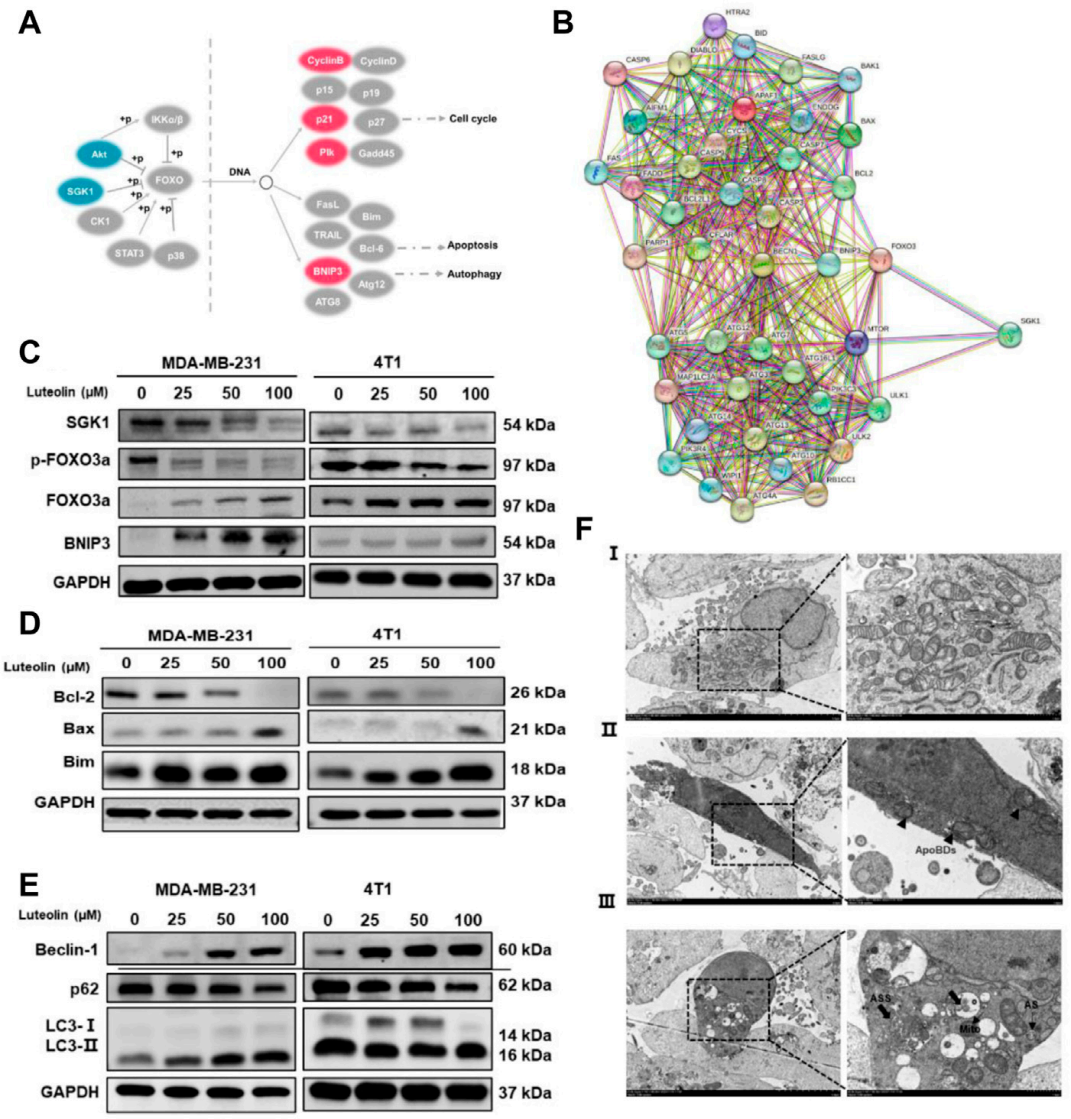
Luteolin encouraged apoptosis and autophagy in triple-negative breast cancer cells via the SGK1-FOXO3a-BNIP3 signaling pathway. **(A)** Differentially expressed genes involved in the FoxO signaling pathway. Red color represents upregulated genes and blue color represents downregulated genes. **(B)** Co-expression analysis of SGK1, FOXO3a, BNIP3, and apoptosis and autophagy-related genes by the string. **(C)** Expression levels of related protein of SGK1-FOXO3a-BNIP3 signaling pathways. **(D)** Expression levels of the apoptosis-related protein. **(E)** Expression levels of the autophagy-related protein. **(F)** The submicroscopic structural morphology was performed by transmission electron microscopy. Scale bar, 5 μm, and 1 μm. All results are represented as the mean ± SEM for at least three independent experiments. *p < 0.05, **p < 0.01, ***p < 0.001, ****p < 0.0001. ApoBDs: apoptotic bodies; AS: autophagosomes; ASS: autophagolysosomes.

Previous studies have shown that SGK1 can phosphorylate FOXO3a and induce apoptosis and autophagy (Liu et al., 2017; Li, 2021). In this study, BNIP3 in the FOXO pathway was significantly upregulated in luteolin-treated MDA-MB-231 cells (Figure 5A), indicating its importance in the regulation of apoptosis and autophagy. In addition, the SGK1-FOXO3a-BNIP3 axis was closely related to the apoptosis gene Bcl-2 and autophagy gene Beclin-1 (Figure 5B), and the expression level of SGK1 and p-FOXO3a decreased as the luteolin concentration increases, while FOXO3a and BNIP3 were significantly upregulated (Figure 5C). Furthermore, related protein expression levels were detected to verify the effect of luteolin on TNBC cell apoptosis, and the results showed that the expression level of Bcl-2, Bax, and Bim increased after being treated (Figure 5D). In addition, the expression levels of autophagy markers Beclin-1 and LC3 Ⅱ/Ⅰ were increased while P62 was decreased (Figure 5E), indicating that luteolin promoted TNBC cell apoptosis. The TME results showed that the shape of partial luteolin-treated cells changed irregularly and presented markedly characteristics of apoptosis, including nuclear chromatin condensation, nuclear fragmentation, and formation of apoptotic bodies (ApoBDs) (Figure 5F). Apart from that, the number of double-membrane structure autophagosomes (AS) and autophagolysosomes (ASS) containing damaged organelles increased, which were typical autophagic changes. In a word, luteolin promoted apoptosis and autophagy of TNBC cells by inhibiting SGK1-p-FOXO3a and promoting FOXO3a-BNIP3, which may be facilitated by the interaction between BNIP3 and Bcl-2/Beclin-1.

### Ectopic expression of SGK1 resists the suppressive effect of luteolin on TNBC cells

**FIGURE 6:**
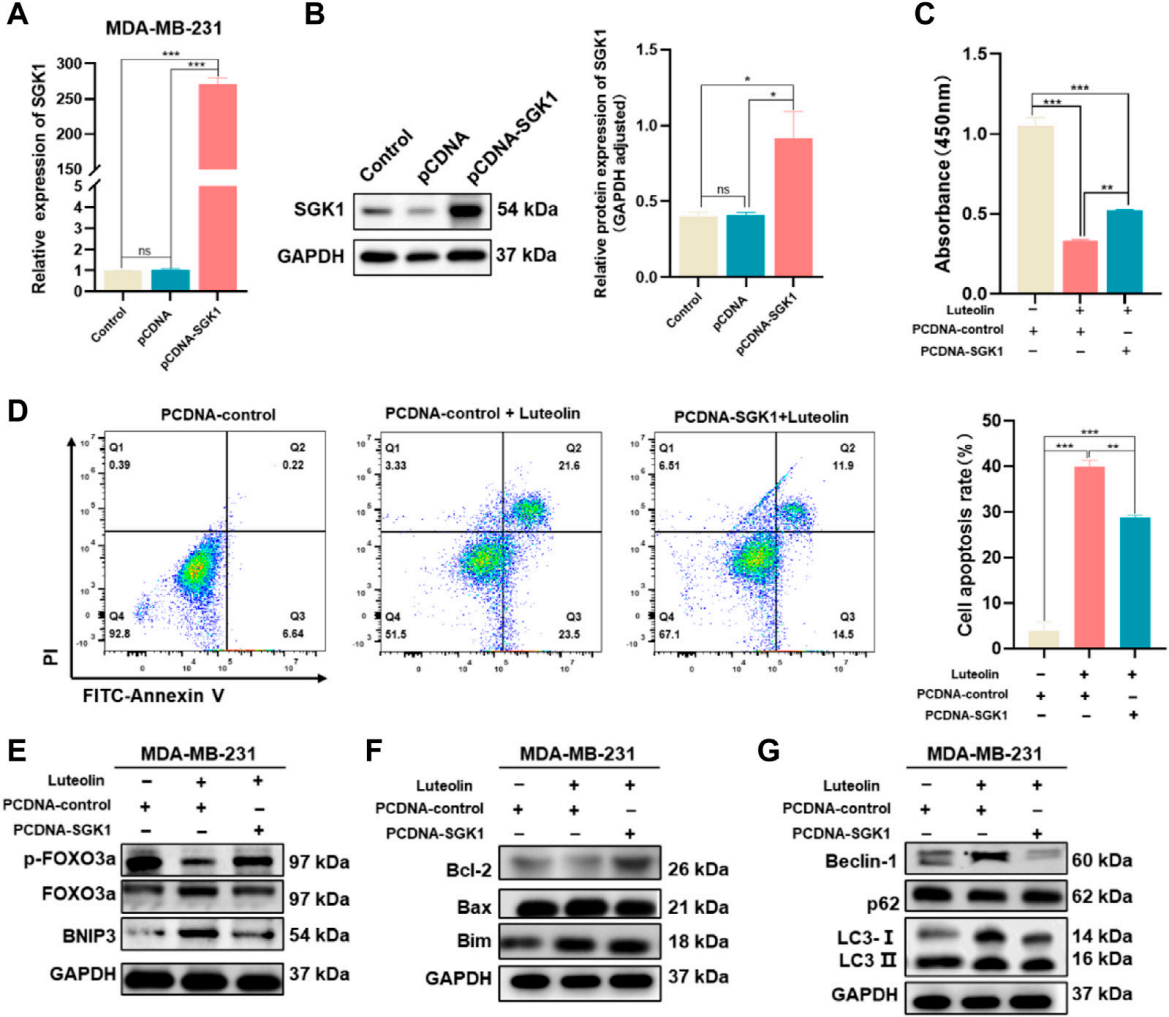
Ectopic expression of SGK1 crippled the suppressive effect of luteolin on TNBC. **(A,B)** Expression of SGK1 was significantly upregulated and examined by RT-qPCR **(A)** and Western blot **(B)**. **(C)** Cell viability analysis. **(D)** Apoptosis activity. **(C)** Expression levels of related protein of SGK1-FOXO3a-BNIP3 pathway. **(D)** Expression levels of the apoptosis-related protein. **(E)** Expression levels of related protein of SGK1-FOXO3a-BNIP3 pathway. **(F)** Expression levels of the apoptosis-related protein. **(G)** Expression levels of the autophagy-related protein. All results are represented as the mean ± SEM for at least three independent experiments. *p < 0.05, **p < 0.01, ***p < 0.0001, ****p < 0.001.

SGK1 expression plasmid (pCDNA-SGK1) was transfected into MDA-MB-231 cells to increase the expression level of SGK1 and the corresponding empty plasmid (pCDNA3.1+) was used as a control (Figures 6A, B). As a result, the cell vitality is stronger, and the phenomenon of apoptosis is less obvious in the experimental group, indicating that the effects of luteolin on cell viability and apoptosis were weakened by the overexpression of SGK1 (Figures 6C, D). In addition, overexpression of SGK1 significantly mitigated the impact of luteolin on the expression levels of the FOXO3a-BNIP3 pathway (Figure 6E). Furthermore, Bcl-2, Bax, Bim, Beclin-1, LC3Ⅱ/Ⅰ, and p62, which belong to the autophagy and apoptosis progress, were all downregulated, indicating that the effects of luteolin on these two signs of progress were attenuated by the overexpression of SGK1 (Figures 6F, G). All these results suggested that SGK1 plays an important role in luteolin-induced apoptosis and autophagy of TNBC cells.

### Luteolin inhibits TNBC growth *in vivo*

**FIGURE 7:**
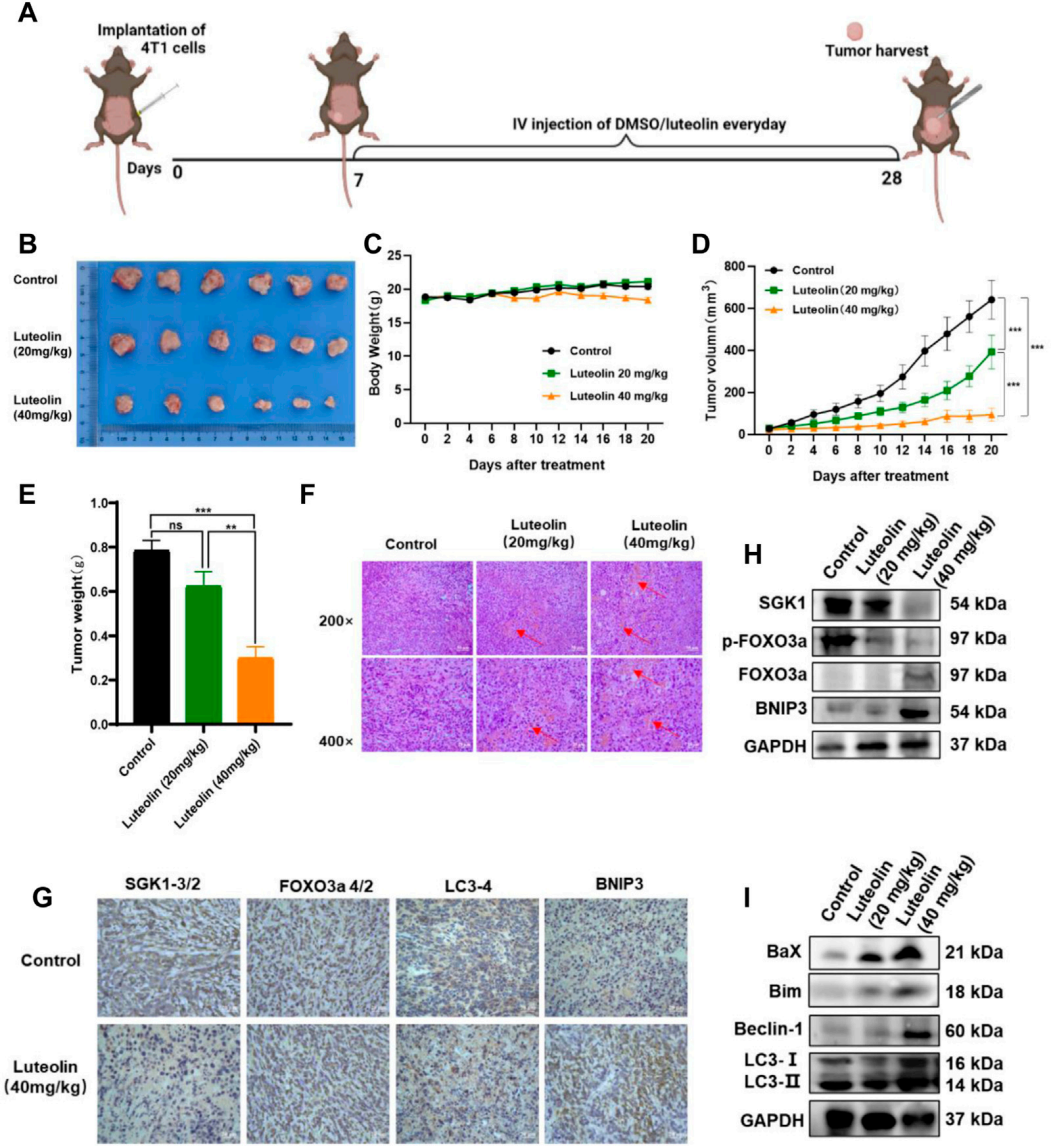
Luteolin decreased the growth of triple-negative breast cancer *in vivo*. **(A)** Orthotropic breast cancer BALB/c mouse mammary model. **(B)** Representative images of tumors. **(C)** Body weight change curves. **(D)** Tumor volume. **(E)** Tumor weight. **(F)** H&E staining images of the tumor. **(G)** Immunocytochemistry analysis of SGK1, FOXO3a, BNIP3, and LC3 expression. **(H)** Expression levels of SGK1, p-FOXO3a, FOXO3a, and BNIP3. **(I)** Expression levels of the autophagy-related protein. All results are represented as the mean ± SEM for at least three independent experiments. *p < 0.05, **p < 0.01, ***p < 0.001, ****p < 0.0001.

To verify the antitumor activity of luteolin on TNBC, a mouse mammary gland model for TNBC-bearing 4T1 cells was constructed (Figure 7A). The implanted tumor tissues were surgically excised and observed (Figure 7B). It was found that luteolin successfully suppressed the volume and weight of the tumor in mice without significant toxicity (Figure 7C). The tumor volume of mice without luteolin treatment was more than 600 mm^3^, while the tumor volume of mice in the low-dose treatment group was around 400 mm^3^, and the tumor volume of the high-dose treatment group was less than 100 mm ^3^ (Figure 7D). At the end of treatment, the weight of dissected tumor also varied considerably among the groups. The tumor weight in the high-dose group was less than 50% of that in the untreated group (Figure 7E). H&E staining demonstrated that luteolin treatment caused evident damage to the tumor, with obvious hemorrhage and increased vacuoles (Figure 7F). Immunohistochemistry analysis demonstrated that SGK1 was downregulated while FOXO3a, BNIP3, and LC3 were upregulated in tumors treated with luteolin (Figure 7G). Western blot confirmed that luteolin resulted in the inhibition of SGK1 and p-Foxo3a and promoted the expression of FOXO3a and BNIP3 (Figure 7H). Additionally, luteolin treatment significantly increased the expression levels of the apoptosis-related proteins Bax and Bim, as well as the autophagy-related proteins LC3-II/Ⅰ and Beclin-1 (Figure 7I). Overall, these findings indicated that luteolin activated both apoptosis and autophagy via the SGK1-FOXO3a-BNIP3 pathway to suppress TNBC growth *in vivo*.

## Discussion

It has been suggested that luteolin inhibited the ability to proliferate and metastasize TNBC cells (Cook et al., 2017). Given the extensive list of luteolin’s pharmacological properties, the studies about its mechanisms are still few. In this study, we verified that luteolin has a superior inhibitory potential toward the proliferation, migration, and invasion of TNBC cells and may prevent tumor development *in vivo*. More notably, we elucidated the underlying mechanisms that luteolin could effectively trigger apoptosis and autophagy in TNBC by inhibiting the phosphorylation of FOXO3a stimulated by SGK1 and promoting BNIP3 expression.

AKT is regarded as an appropriate target for cancer treatment because of its propensity to accelerate tumor progression and resistance to a variety of chemotherapeutic agents (Ma et al., 2019; Gallyas et al., 2020; Sobočan et al., 2020). However, a large proportion of patients have poor sensitivity to various single-agent Akt inhibitors including GSK2110183 (Spencer et al., 2014), capivasertib (AZD5363) (Robertson et al., 2022), and GDC-0068 (Ippen et al., 2019) which are being evaluated in clinical trials currently. Furthermore, there is proof that the combination of AGC inhibitors may boost the pertinent effectiveness (Prêtre and Wicki, 2018). In this study, Luteolin could dramatically affect the biological processes apoptotic in TNBC cells, and the PI3K-Akt signaling pathway was the most significant DEGs enriched associated pathway. Luteolin significantly inhibits not only AKT3 but also SGK1, which is a member of AGC kinases and shares roughly 54% of its catalytic domain with the major cellular survival factor, protein kinase B (PKB, also called Akt), indicating structurally and functionally analogous (Kobayashi et al., 1999). In agreement with previous studies, overexpression of SGK1 greatly reduced the inhibitory effects of luteolin on TNBC cell growth, indicating that targeting both SGK1 and AKT is more effective in suppressing cell growth than inhibiting AKT alone (Orlacchio et al., 2017). Even more interesting is that docking simulations between luteolin and AKT/SGK1 indicated that luteolin has a better interaction with SGK1, owing to which SGK1 was thought to be a crucial part of the AKT-independent pathway for PI3K-mediated TNBC proliferation.

The TME results showed that luteolin-treated cells presented markedly characteristics of apoptosis and autophagy. Furthermore, in the FOXO pathway, luteolin upregulated the expression of FOXO3a target gene BNIP3 (Bcl-2/adenovirus E1B 19-kDa interacting protein) (Figure 5A), which is a pro-apoptotic BH3-only protein of the Bcl-2 family inducing both apoptosis and autophagy characteristics (Niu et al., 2019; Madhu et al., 2023). Phosphorylated SGK1 is the phospho-donor of the FOXO3a (Guo et al., 2013) and the phosphorylation of FOXO3a (p-FOXO3a) creates 14-3-3 protein docking sites which leads it to lose its nuclear localization and transcriptional activity (Xing et al., 2021). Cytoplasmic p-FOXO3a is further ubiquitinated and then subjected to degradation in a ubiquitin/proteasome-dependent way (Wang et al., 2015). Thus, we hypothesize that luteolin-mediated SGK1 inhibition may promote apoptosis and autophagy via the FOXO3a-BNIP3 axis. To confirm our hypothesis, the expression levels of related proteins, including SGK1, FOXO3a, p-FOXO3a, and BNIP3 in two TNBC cell lines and xenograft tumors, were confirmed with the interaction of different concentrations of luteolin. The results showed that luteolin could cause downregulation of p-FOXO3a, and upregulation of FOXO3a and BNIP3 by eliminating the expression level of SGK1. Those findings suggest that SGK1 restriction induced by luteolin facilitates the export of FOXO3a from the cytoplasm to the nucleus by preventing FOXO3a phosphorylation and degradation, consequently upregulating BNIP3 expression.

To validate the promotion of luteolin on apoptosis and autophagy in TNBC, we demonstrated that BNIP3 regulates apoptosis and autophagy by interacting with Bcl-2 and autophagy markers LC3. The balance of controlling the family of Bcl-2 proteins consisting of pro-apoptotic versus anti-apoptotic members has a central function in the regulation of apoptosis (Kuwana and Newmeyer, 2003; Delbridge et al., 2016). BH3-only pro-apoptotic proteins (Bid, Bim, Bad, Puma, and Noxa) can be grouped as activators that can bind and directly activate pro-apoptotic protein Bax and/or Bak(Galonek and Hardwick, 2006; Adams and Cory, 2007), and sensitizer proteins which can inhibit anti-apoptotic proteins (Bcl-2, Bcl-XL, Bcl-W, and Mcl1) and activate Bax and/or Bak indirectly (Rizzuto and Pozzan, 2006; Jozefczuk et al., 2020). We further confirmed luteolin significantly suppressed Bcl-2 and increased Bax, indicating that BNIP3 can bind to Bcl-2 and form heterodimers to activate pro-apoptotic proteins, Bax, eventually resulting in apoptosis. In addition, some studies suggest that FOXO3a can activate apoptotic pathways by directly binding to the promoter region of the Bim gene to induce its expression (Patra et al., 2021). In line with past studies, we also verified that luteolin could affect the Bim expression. In the progress of autophagy, LC3-I is modified and transformed by ubiquitin to produce LC3-II, which is involved in the formation of autophagosomes and is a marker for autophagy (Peña-Martinez et al., 2022). LC3-interacting region motifs found in the cytosolic N-terminal domains of BNIP3 and processed LC3 at phagophores allow for interactions between the two proteins (Hanna et al., 2012). Bcl-2 has also been shown to suppress autophagy by binding and neutralizing Beclin-1 through its BH3 domain (Shimizu et al., 2004; Maiuri et al., 2007). Compared to the control group, Western blot analysis showed that the levels of Beclin-1and LC3-II/LC3-Ⅰ in TNBC treated with luteolin were markedly increased, while the autophagy receptor protein p62 level was decreased. Notably, the crucial of luteolin in regulating autophagy in TNBC cells depends on the inhibition of SGK1 and activation of BNIP3 downstream. It proves the advantageous administration of luteolin in TNBC therapy and one of the mechanisms luteolin exploits to encourage TNBC cell death seems to be by activating autophagy. However, as a strictly controlled catabolic progress, autophagy plays a dual role in cancer initiation and progression. The association between autophagy and apoptosis is unrevealed and still needs to be explored in depth.

**FIGURE 8:**
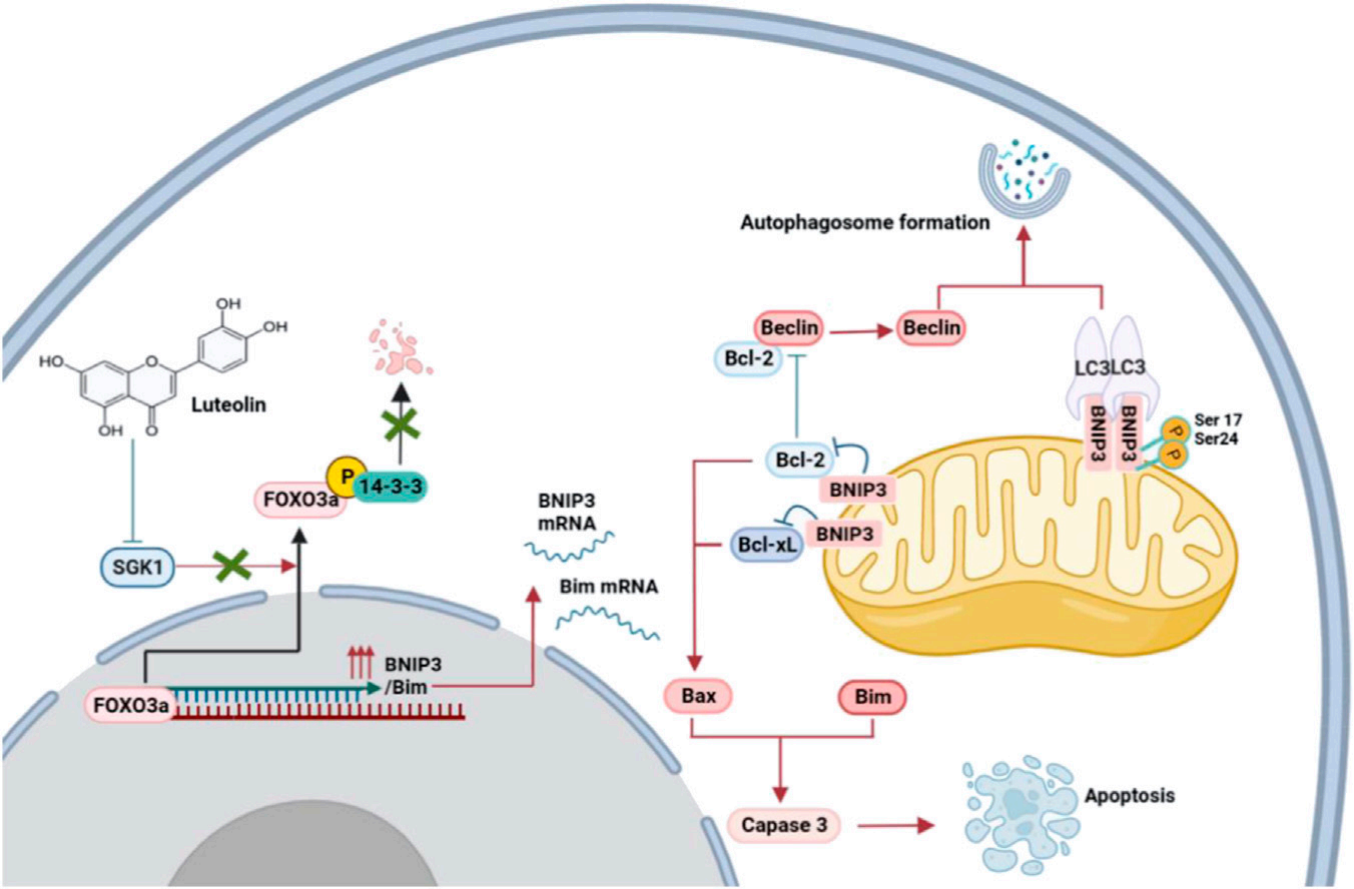
Proposed mechanisms of the inhibition of luteolin in TNBC via SGK1-FOXO3a-BNIP3 signaling. Naturally, FOXO3a is located in the nucleus, where it attaches to DNA and dynamically modulates the expression of genes. The phosphorylation of FOXO3a creates 14-3-3 protein docking sites which leads it to lose its nuclear localization and transcriptional activity. Cytoplasmic p-FOXO3a is further ubiquitinated and then subjected to degradation. The inhibition of SGK1 by luteolin suppresses the phosphorylation and degradation of FOXO3a, which results in the upregulation of target genes BNIP3 and Bim and eventually induces apoptosis and autophagy.

It is obvious that the mechanism of SGK1 inhibition-induced BNIP3 elevation may be dependent on the release of Bax and Bim to stimulate apoptosis and Beclin-1 to trigger autophagy (Figure 8). To determine whether SGK1 was the main target of luteolin to inhibit TNBC, we generated SGK1 overexpression TNBC cells. The ectopic expression of SGK1 significantly alleviated the inhibitory effects on TNBC cells mediated by luteolin and the autophagy and apoptosis were restricted, indicating that SGK1 is essential for the control of cell death. We believe that luteolin significantly contributes to the suppression of SGK1, which might potentially serve as a feasible therapeutic target in TNBC progression, and help us develop more attractive therapeutic approaches in future clinical trials.

## Conclusion

In summary, we demonstrated that luteolin regulated apoptosis and autophagy by targeting SGK1. Meanwhile, the reintroduction of FOXO3a into the nucleus and the upregulation of BNIP3 is critical for the blockade of SGK1-induced autophagy and apoptosis. Here we report, for the first time, that luteolin has a promotion on apoptosis and autophagy by the SGK1-FOXO3a-BNIP3 pathway in TNBC, which provides novel insights into the exploring of anti-cancer efficacy of luteolin and implies that SGK1 might provide a therapeutic target for TNBC.

## Data Availability

The datasets presented in this study can be found in online repositories. The names of the repository/repositories and accession number(s) can be found in the article/Supplementary Material.

## Abbreviations

AS, autophagosomes; ASS, autophagolysosomes; DEGs, differentially expressed genes; FOXO, forkhead box O; poBDs, apoptotic bodies; SGK1, Serum and glucocorticoid-induced protein kinase 1; TNBC, Triple-negative breast cancer.
