# Supplementary material for: Luteolin inhibits triple-negative breast cancer by inducing apoptosis and autophagy through SGK1-FOXO3a-BNIP3 signaling
Source: Front Pharmacol. 2023 Jun 6;14:1200843. doi: 10.3389/fphar.2023.1200843 (PMC10279868; doi:10.3389/fphar.2023.1200843)
Supplement: Supplementary file 1 [file Presentation1.pdf]

Supplementary Figure S1

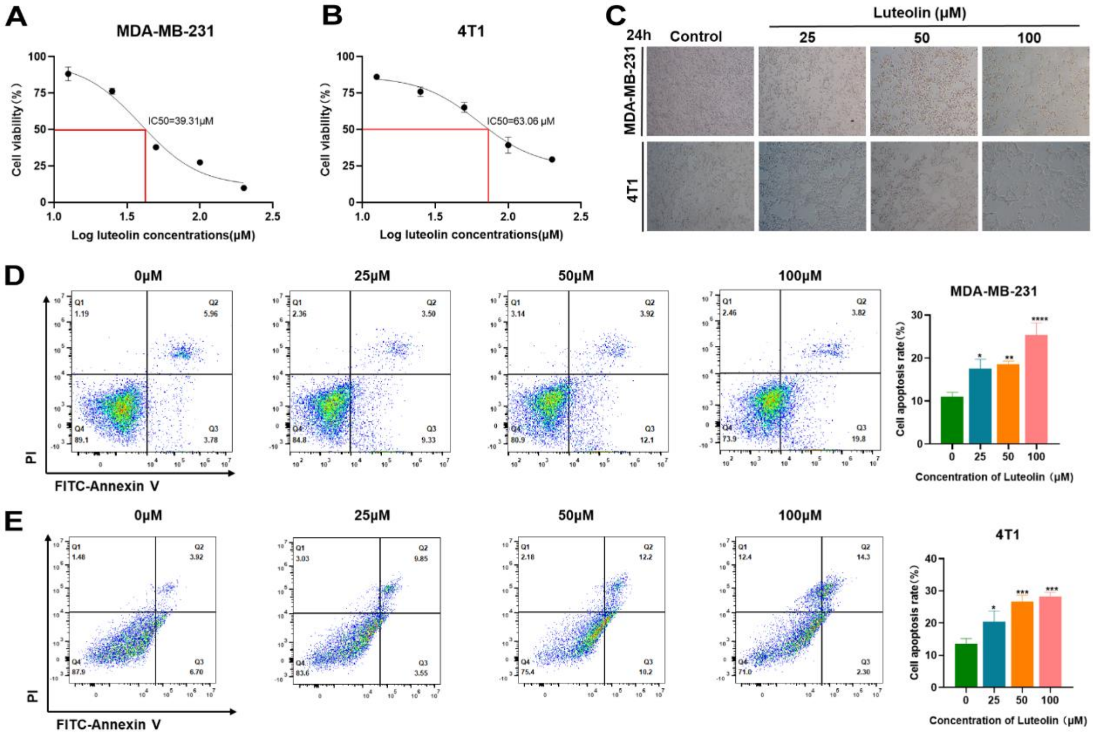

Figure S1. Luteolin inhibited triple-negative breast cancer cell viability and promoted apoptosis.

（**A-B**）The half-maximal inhibitory concentration values of luteolin treated for 48 h for MDA-MB-231 cells (A) and 4T1 cells (B) in a time and dose-dependent manner. **(C)** The morphological features of MDA-MB-231 and 4T1 cells treated with luteolin for 24 h. **(D-E)** Apoptosis activity of MDA-MB-231 cells (D) and 4T1 cells (E) treated with luteolin for 24 h. All results are represented as the mean ± SEM for at least three independent experiments. * $p < 0.05$, ** $p < 0.01$, *** $p < 0.001$
