# Supplementary material for: Luteolin inhibits triple-negative breast cancer by inducing apoptosis and autophagy through SGK1-FOXO3a-BNIP3 signaling
Source: Front Pharmacol. 2023 Jun 6;14:1200843. doi: 10.3389/fphar.2023.1200843 (PMC10279868; doi:10.3389/fphar.2023.1200843)
Supplement: Supplementary file 3 [file Presentation2.pdf]

Supplementary Figure S2

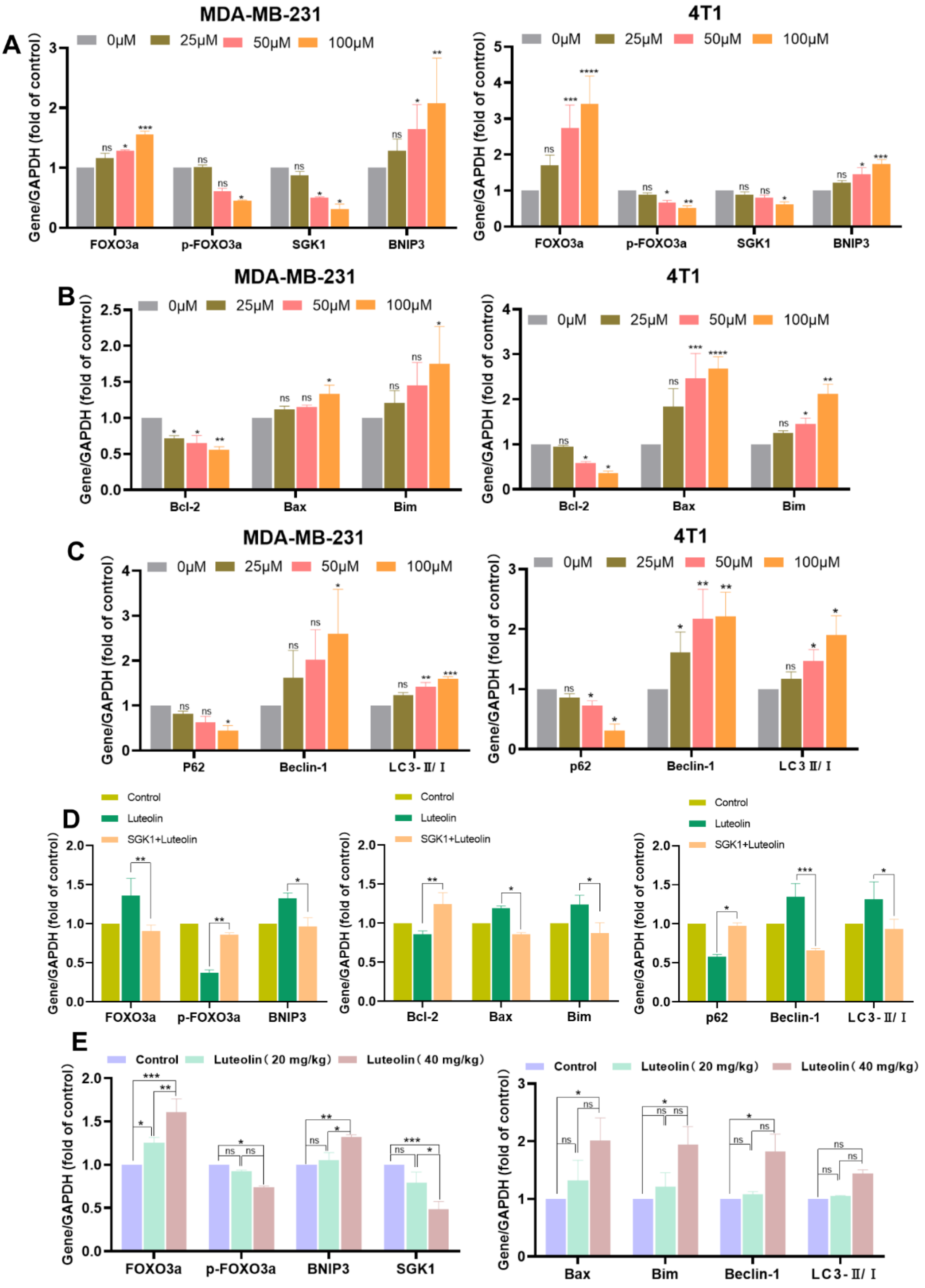

Figure S2. Quantification of western blot.(A) Expression levels of related protein of SGK1-FOXO3a-BNIP3 signaling pathways. **(B)** Expression levels of the apoptosis-related protein. **(C)** Expression levels of the autophagy-related protein .**(D)**Expression leves of the related protein in cells overexpressing SGK1. **(E)** Expression leves of the related protein in tumor. *$p < 0.05$, **$p < 0.01$, ***

$p < 0.001$
